# Whole genome sequencing-based characterization and determination of quinolone resistance among methicillin-resistant and methicillin-susceptible *S. Aureus* isolates from patients attending regional referral hospitals in Tanzania

**DOI:** 10.1186/s12864-024-11045-z

**Published:** 2024-11-22

**Authors:** Masoud A. Juma, Happiness Kumburu, Boaz Wadugu, Davis Kuchaka, Mariana Shayo, Patrick Kimu, Livin E. Kanje, Melkiory Beti, Marco van Zwetselaar, Blandina Mmbaga, Tolbert Sonda

**Affiliations:** 1 Kilimanjaro Christian Medical University College (KCMUCo), Kilimanjaro, Tanzania; 2 Kilimanjaro Clinical Research Institute, Kilimanjaro, Tanzania; 3 Department of Microbiology and Parasitology, State University of Zanzibar (SUZA), Zanzibar, Tanzania; 4 Muhimbili University of Health and Allied Sciences (MUHAS), Dar es Salaam, Tanzania

**Keywords:** *Staphylococcus* Cassette chromosome mec (SCC*mec*), Sequence type (ST), Staphylococcal protein A (*spa*), Methicillin-resistant *S. Aureus* (MRSA)

## Abstract

**Background:**

The emergence of multidrug-resistant termed Methicillin-resistant *Staphylococcus aureus (*MRSA) strain, driven by the acquisition of resistance gene *mecA* imposes a substantial challenge in the treatment and control of their related infections. Although quinolones have historically been effective against both MRSA and methicillin-susceptible *S. aureus* (MSSA) strains, the rising resistance to quinolones among *S. aureus* isolates, particularly in MRSA, has severely curtailed their potency and further narrowed down the therapeutic options. This study aimed to determine the burden of MRSA among isolates, as well as their resistance profile, genotypic characterization, and molecular relatedness through the construction of a phylogenetic tree.

**Materials and methods:**

Archived clinical *S. aureus* isolates from a descriptive, cross-sectional study involving six regional referral hospitals in Dodoma, Songea, Kigoma, Kitete, and Morogoro in the mainland Tanzania and Mnazi Mmoja in Zanzibar were analyzed. Bacterial identification was performed using both classical microbiology and whole genome sequencing on Illumina Nextseq 550 Sequencer. Species identification was done using KmerFinder 3.2, Multilocus Sequence Typing using MLST 2.0, SCC*mec* typing using SCCmecFinder 1.2, resistance genes using ResFinder 4.1, and phylogenetic relatedness using CSI Phylogeny 1.4.

**Results:**

Out of the 140 isolates analyzed, 69 (49.3%) were identified as MRSA, with 57 (82.6%) exhibiting quinolone resistance. Conversely, 71 isolates were identified as MSSA, and none of them exhibited resistance to quinolones. *Spa*-typing revealed six spa types, with t355, t1476, and t498 being the most common. Moreover, all (69) MRSA were found to carry SCC*mec* type IV. The isolates exhibited 14 different sequence types (STs). Notably, ST152 was prevalent among MSSA (50 isolates, 70%), while ST8 was the predominant sequence type among MRSA (58 isolates, 84%). The antimicrobial resistance profile revealed at least three horizontally acquired resistance genes, with *blaZ*, *dfrG*, *tet(K)*, and *aac (6’)-aph (2’’)* genes being highly prevalent.

**Conclusion:**

There is a high genetic diversity among the *S. aureus* isolates existing in Tanzania regional hospitals, with a concerning burden of quinolone resistance among MRSA isolates. The diversity in resistance genes among MRSA lineages emphasizes the necessity for the development of sustainable antimicrobial stewardship and surveillance to support evidence-based guidelines for managing and controlling MRSA infections in both community and hospital settings.

## Introduction

*Staphylococcus aureus* has transitioned into a formidable opportunistic pathogen capable of causing clinical infections ranging from mild to life-threatening [1]. The virulence of this bacterium is based on its ability to secrete enzymes and an array of potent toxins, promoting tissue invasion and diseases such as food intoxication, scalded skin syndrome, and toxic shock syndrome [1, 2]. A pivotal milestone in this evolutionary arms race is the horizontal acquisition of the *mecA* gene carried within the SCC*mec* mobile genetic element, transforming *S. aureus* into a multidrug-resistant strain known as Methicillin-resistant *S. aureus* (MRSA) [3]. This resilient strain has established itself as a public health concern due to its remarkable ability to cause life-threatening infections and its propensity to swiftly spread between human hosts [2, 4]. At the molecular level, the *mecA* gene encodes a unique penicillin-binding protein-2a (PBP2a), an enzyme with a remarkably low affinity to β-lactam antibiotics and thus confer resistance to broad classes of antibiotics such as penicillin, cephalosporins, carbapenems, and monobactams [3, 5]. Furthermore, the SCC*mec* elements can house resistance genes for other non-β-lactam antibiotics such as macrolides, aminoglycosides, and clindamycin [6–9]. This further exacerbates the difficulty of treating MRSA strains, consequently making it one of the most successful pathogens causing infections accompanied by high morbidity, mortality, prolonged hospital stays, and treatment failure [6, 10–12]. Such dire consequences prompted the World Health Organization to prioritize MRSA at the top of the high-priority list of bacteria in 2017, compelling the scientific community to seek novel antibacterial agents [12, 13]. Nevertheless, the significance of *S. aureus* extends beyond MRSA, as methicillin-susceptible *S. aureus* (MSSA) strains remain medically relevant and capable of causing serious clinical infections. However, the absence of the *mecA* gene renders this strain susceptible to a wide variety of β-lactams and less virulent [14]. As the efficacy of β-lactams antibiotics has waned, alternative agents such as quinolones gained prominence for MRSA-related infections, offering a broad spectrum activity and reduced toxicity compared to vancomycin [15, 16]. Yet, the increasing quinolone resistance in *S. aureus* isolates has severely hampered the usefulness of these agents, further narrowing the treatment options [17].

Like any other bacteria, *Staphylococcus aureus* has developed multiple antibiotic resistance mechanisms, including gene acquisition, efflux pumps, enzymatic deactivation, and target site mutations, with the latter being the primary mechanism for quinolones resistance [18, 19]. Quinolones inhibit DNA gyrase (encoded by the *gyrA* gene) and Topoisomerase IV (encoded by the *grlA* gene), enzymes crucial for bacterial DNA replication [20, 21]. However, in *S. aureus* topoisomerase IV is the primary target, while DNA gyrase serves as a secondary target regarding quinolone resistance [10, 22]. Mutations in these genes occur in Quinolone-resistance determining regions (QRDR), specific regions whose mutations lead to quinolone resistance [10]. Curiously, mutations in this region change the amino acids and lower the affinity of the enzyme-DNA complex to quinolones, resulting in resistance [10, 17]. Dual mutations in *grlA* (codons 79, 80) and *gyrA* (codons 80, 84, 116) are linked to elevated quinolone resistance [10, 23]. A single point mutation in the *grlA* gene alone can confer a high level of quinolone resistance while isolated mutations in *gyrA* or overexpression of the *norA* efflux pump contribute to a lower resistance level [23].

In Tanzania, where the burden of MRSA is substantial, the detection of drug resistance and screening for MRSA primarily relies on phenotypic methods [24]. This consequently results in an underestimation of the MRSA burden, creating a knowledge gap in comprehending the genomic and evolutionary characteristics that underlie the successful adaptation and persistence of *S. aureus* in both hospitals and community settings. This study, therefore, aimed to bridge these knowledge gaps by harnessing whole genome sequencing for a comprehensive analysis of clinical *S. aureus* isolates. Thus, it provides critical insights into this pervasive and evolving bacterium by providing molecular epidemiology, genotypic characterization, detection of resistance profiles, and exploration of molecular relationships among the studied *S. aureus* isolates.

## Materials and methods

### Study design and sites

**Fig. 1:**
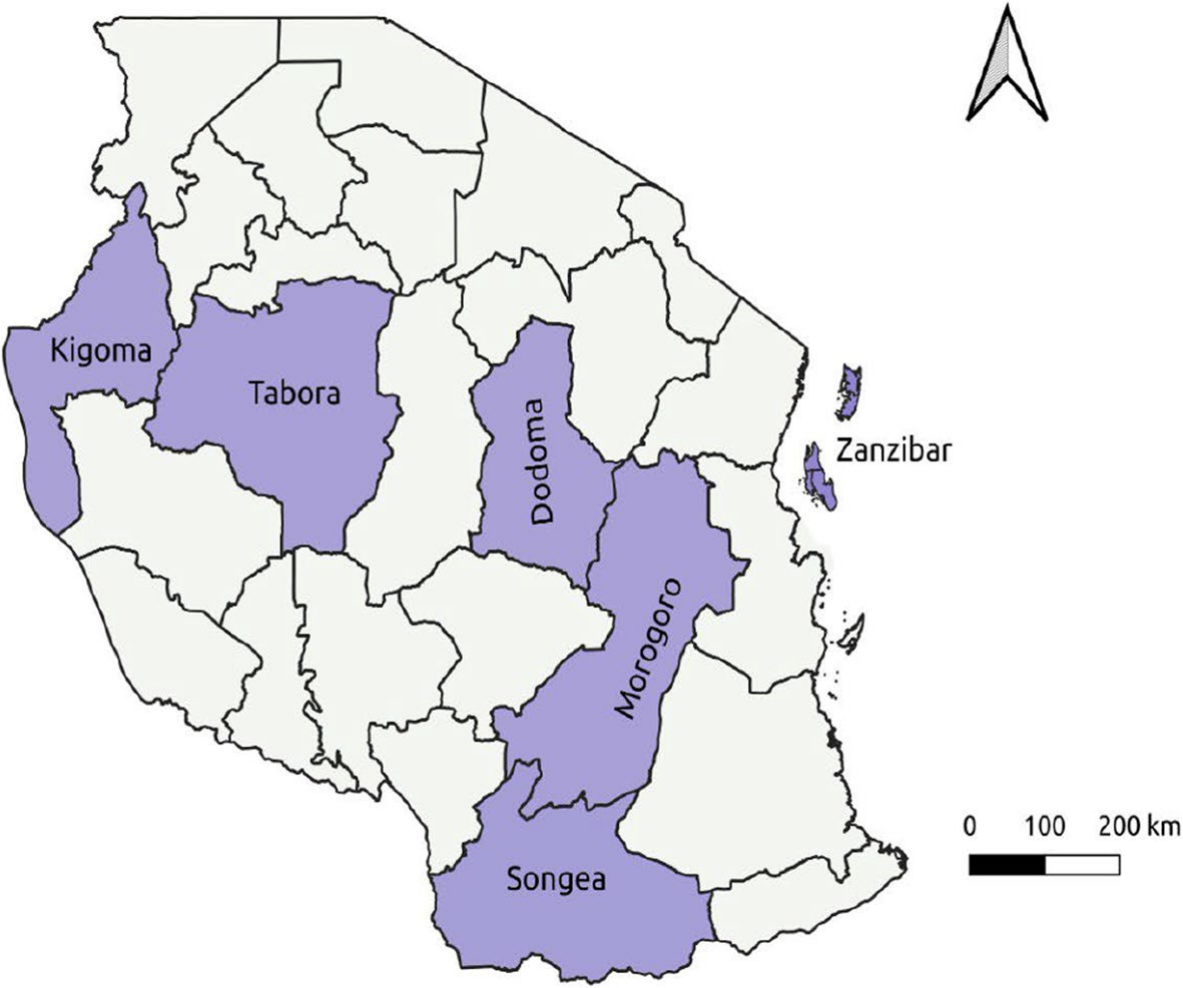
A map of Tanzania showing the geographical locations of the six regions whose regional referral hospitals were included in this study

This descriptive, cross-sectional study utilized archived *S. aureus isolates* collected from clinical samples of both inpatients and outpatients of all age groups attending regional referral hospitals in Tanzania from January 2020 to December 2021. These included Tabora, Dodoma, Songea, Kigoma, Morogoro Regional Referral Hospitals from mainland Tanzania, and Mnazi Mmoja Referral Hospital from Zanzibar. For inpatient participants to be included in the study, they were supposed to be admitted within 48 h before the sample collection procedure. All archived *S. aureus* isolates collected from January 2020 to December 2021 with complete sets of demographic and cultural result information were included. Isolates with missing demographic information were excluded. The isolates were recovered from culture growth of pus, urine, blood, and cerebrospinal fluid (CSF). The geographical locations of the regions from which isolates were taken are shown in the map below (Fig. 1).

### Subculturing, DNA extraction, library preparation, and sequencing

The isolates were retrieved from − 80^o^C, allowed to attain room temperature before being subcultured in Blood Agar media, and incubated overnight at 35^o^C. Species identification was done using both phenotypic and genotypic approaches. Phenotypic re-identification was performed using gram stain, catalase, and coagulase tests respectively. Genotypic identification was performed based on whole genome sequencing. The DNA extraction was done using Quick-DNA™ Fungal/Bacteria Miniprep Kit per the manufacturer’s instructions. Following extraction, DNA quantification was done using a Qubit^®^ version 4.0 fluorometer. Library preparation was performed based on the NEBNext^®^ Ultra™ II FS DNA Library Prep Kit manual and Illumina Nextseq550 next-generation sequencer (2 × 250 bp paired-end reads protocol). Briefly, library preparation involves fragmentation of DNA, adaptor ligation, size selection, barcoding, and pooling of each extracted genomic DNA. After preparation, the library was normalized and mixed with a PhiX control, then loaded onto the Illumina Nextseq 550 sequencer for DNA sequencing.

#### Bioinformatics analysis

Trimming of adapters and filtering of the short reads was done using a Trimmomatic tool 0.39. Quality control of the sequenced raw reads was also done using FastQC 0.12.0. Furthermore, De novo assembly was performed using St. Petersburg genome assembler (SPades) 3.15.5, whereas the final output files in Fasta format containing several contigs were obtained. The sequence data were then further analyzed using CGE tools found in a Bacterial Analysis Pipeline (BAP 3.3.2), online available at www.genomicepidemiology.org. The species identification of the sequenced isolates was determined using KmerFinder 3.2 [25]. Multilocus Sequence typing based on sequencing seven housekeeping genes (*pta*, *arcC*, *tpi*, *aroE*, *gmk*, *yqiL*, and *glpF*), and Sequence types (STs) were determined using MLST 2.0 [26]. Furthermore, resistance genes, virulence genes, and SCC*mec* type were also determined by using ResFinder 3.2, virulenceFinder 2.0, and SCC*mec* Finder 1.2 respectively [27–30]. The *Staphylococcus* protein A type (spa-type) was determined by using spaTyper 1.0. Again, for analysis of interstrain whole genome single nucleotide polymorphism (SNPs), CSI Phylogeny 1.4 was used [24, 26]. The tool generated Newick files. For further annotation and visualization of the phylogenetic tree, Figtree 1.4.4 software was used [26]. The whole genome SNPs tree included whole genomes of 57 sequenced MRSA isolates, and *S. aureus* US500 (accession number CP000255) was used as a reference genome. For the detection of chromosomal point mutations leading to quinolone resistance, PointFinder, part of ResFinder 4.3.0 was used [27]. The presence of the *mecA* gene in the genome was used to categorize the isolate as Methicillin-resistant *Staphylococcus aureus*.

#### Statistical analysis

SPSS version 20 was used to analyze and summarize the demographic and culture results data using frequency and proportions. The chi-square test was used to check for statistical association, and a *p*-value of < 0.05 was considered statistically significant.

## Results

### Clinical and demographic characteristics of the isolates

A total of 140 isolates confirmed as *S. aureus* bacteria by both phenotypic and genotypic methods were included in this study. The isolates were unevenly distributed across the six regional referral hospitals. Of the 140 isolates, Dodoma, Morogoro, and Mnazi Mmoja Referral Hospitals contributed 49 (35%), 36 (25%), and 31 (22%) respectively. The other three Referral Hospitals, namely Tabora, Songea, and Kigoma, contributed 15 (10%), 5 (3.5%), and 4 (2.8%) isolates, respectively. Again, the majority of the isolates (55.7%) were collected from Inpatients while only (44.3%) were from Outpatients. Of the 140 *S. aureus* isolates, the majority, (63.6%), were recovered from patients’ wound pus, followed by urine (16.4%), blood (14.3%), and sputum (2.1%). It is worth noting that, most of these patients (56%) had septic wound infection as a major underlying medical condition (Table 1).

**Table 1:** Clinical and demographic characteristics of the isolates

	Frequency (*n*)	(*n*/*N*) %
**Sample source**		
Pus	89	**(63.6)**
Urine	23	(16.4)
Blood	20	(14.3)
HVS	4	(2.9)
Sputum	3	(2.1)
Stool	1	(0.7)
**Sex**		
Male	74	**(52.9)**
Female	66	(47.1)
**Age groups**		
Adult (≥ 18-year-old)	123	**(87.9)**
Children (< 18-year-old)	17	(12.1)
**Departments**		
Surgical	61	**(43.6)**
Medical ward	36	(25.7)
OPD	16	(11.4)
Pediatric	15	(10.7)
Gynecology	7	(5.0)
Orthopedic	5	(3.6)
**Type of patients**		
Inpatient	78	**(55.7)**
Outpatient	62	(44.3)
**Underlying medical conditions**		
Septic wound infection	77	**(56.4)**
Genitourinary infection	28	(20.0)
Septicemia	19	(13.6)
Skin infection	10	(7.1)
Gastritis	1	(0.7)
**Total**	**140**	**100**

*HVS* High Vaginal Swab, *OPD* Outpatient Department

### The proportion of MRSA and MSSA with quinolone resistance

**Fig. 2:**
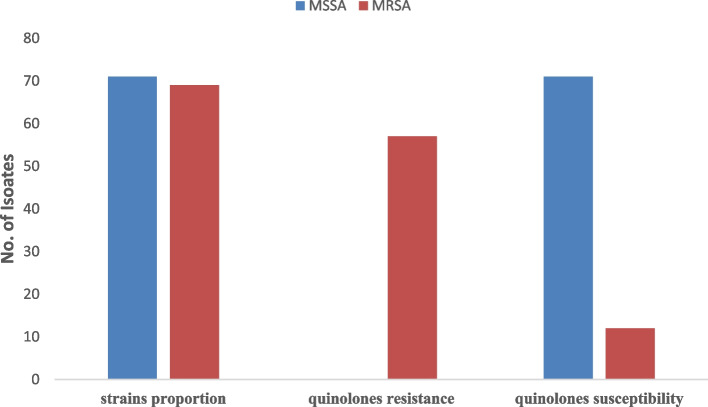
Proportion of MRSA/MSSA with their Quinolones resistance/susceptibility relationship

From a total of 140 isolates sequenced, 69 (49.3%) were found harboring the *mecA* gene (therefore MRSA), and 71 (50.7%) were found as MSSA (no *mecA* gene). The presence of the *mecA* gene was phenotypically confirmed using cefoxitin disk diffusion (30 µg), whereas all isolates containing the *mecA* genes demonstrated resistance to cefoxitin with an inhibition zone of ≤ 21 mm. For quinolones resistance, all (71) MSSA isolates were susceptible to quinolones, i.e., ciprofloxacin/nalidixic acid, while the majority of MRSA isolates, 57 (82.6%) exhibited resistance to quinolones (Fig. 2).

### Mutations conferring Quinolone’s resistance and levels of quinolone resistance

No quinolone resistance genes were found in any of the isolates exhibiting resistance to quinolones. The observed resistance to quinolones was mediated by a combination of single-point mutations that occurred at different positions in two non-resistance genes: *grlA* (p.S80Y) and *gyrA* (p.S84L). Nonetheless, no point mutation in either *grlB* or *gyrB* genes was found. No other quinolone resistance determinants were identified across the isolates.

### Genotypic characterization of the MRSA/MSSA isolates

**Fig. 3:**
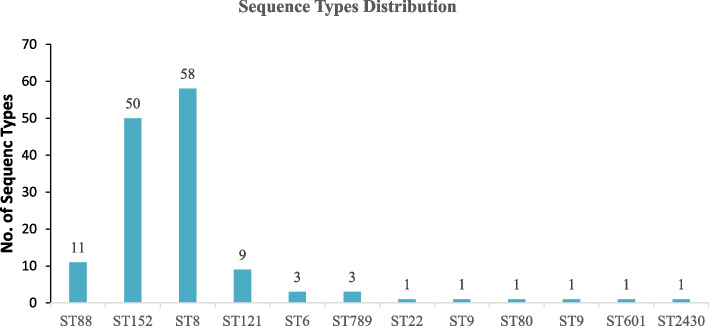
Distribution of Sequence Types (ST) among the *S. aureus* isolates

MLST identified 12 different sequence types (STs). ST8 was the most prevalent (*n* = 58), followed by ST152 (*n* = 50) and ST88 (*n* = 11). All ST8 isolates were MRSA, while those belonging to ST152 were MSSA. A few isolates with ST88 were also identified as MRSA strains (Fig. 3). Predominantly linked to MSSA, ST152 strains may represent a less virulent subset. Their susceptibility to methicillin suggests potential treatment options. ST8, this sequence type was the most prevalent and exclusively associated with MRSA. ST8 strains are often clinically relevant and have been implicated in healthcare-associated infections.

**Fig. 4:**
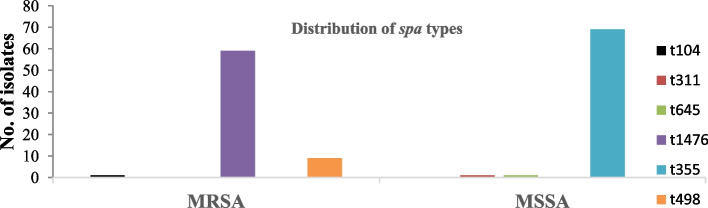
Distribution of *Spa-*types among the MRSA and MSSA bacterial isolates

The predominant *spa* types were t355 69 (49%), t1476 59 (42%), and t498 9 (6.4%) respectively. SCC*mec* typing revealed that all MRSA isolates carried SCC*mec* type IV elements. The predominant genotype among MSSA isolates was identified as ST152-*spa*-t355, while ST8-SCC*mec*IV-*spa*-t1476 was predominant among MRSA isolates (Fig. 4). Additionally, 14 (20%) of these isolates were positive for Panton-Valentine Leucocidin (*Pvl*) genes, which encode for Panton-Valentine Leucocidin toxins.

### Distribution of resistance genes among the MRSA and MSSA isolates

**Fig. 5:**
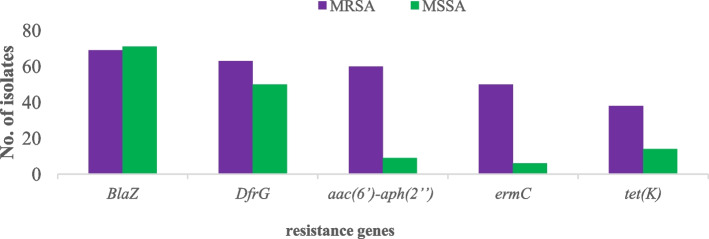
Distribution of resistance genes among the *S. aureus* isolates

A vast array of resistance genes was detected among the isolates in this study. The most prevalent detected resistance genes were *blaZ* which confers resistance to penicillin, *dfrG* which confers resistance to trimethoprim, *aac (6’)-aph (2’’)* which confers resistance to aminoglycosides, *mecA* which confers resistance to broad classes of β-lactam antibiotics, *ermC* which confers resistance to macrolides, and *tet(K)* gene which confers resistance to tetracycline. The aforementioned resistance genes had frequency distribution of 140 (100%), 113 (81%), 69 (49.3%), 69 (49.3%), 66 (47%), and 51 (36%) respectively. It was notably observed that MRSA isolates harbored most of these aforementioned genes compared to MSSA isolates. Remarkably, the *blaZ* gene, demonstrated a uniform distribution across all *S. aureus* isolates, irrespective of their MRSA or MSSA classification (Fig. 5). Different resistance gene combinations were detected throughout the isolates such as *blaZ*-dfrG, *aac (6’)-aph (2’’)*-*blaZ*-*dfrG*-*ermC*-*mecA* and *aac (6’)-aph (2’’)*-*blaZ*-*dfrG*-*ermC*-*mecA*-*tet(K)*. These resistance gene combinations account for 20.7%, 17%, and 10% respectively.

### Phylogenetic relationship (SNPs) among MRSA isolates exhibiting quinolone resistance

**Fig. 6:**
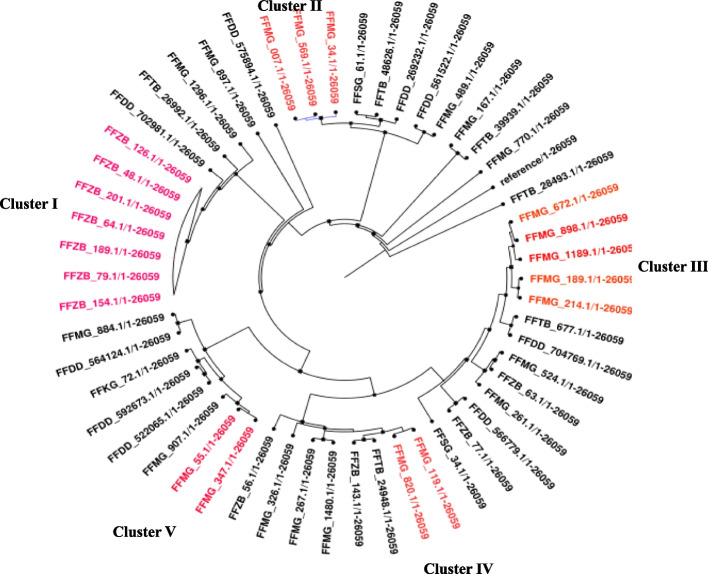
Phylogenetic tree of MRSA isolates with quinolone resistance

To identify SNPs, a total of 55 MRSA isolates exhibiting quinolone resistance were mapped against a reference genome of *S. aureus* US300 FPR3757 (accession number CP000255, chromosome length 2,917,469 bp). The average percentage of reference genome covered by all isolates was 93.4%, with 2,270,301 positions covered with SNP differences ranging from 3 to 1,9706. The SNP analysis of 55 MRSA isolates with quinolone resistance revealed a difference in SNP from 4 to 1,3539. Importantly, five clusters in MRSA isolates with SNP differences of less than 25 were observed. These clusters had a combination of ST8, SCC*mec*IV, and *spa* type 1476 except for one clade from Mnazi Mmoja hospital, whereas the isolates had a combination of ST88, SCC*mec* IV, and *spa* type 4968 (Fig. 6).

## Discussion

*S. aureus*, a prominent gram-positive pathogen, is responsible for a wide variety of diseases encompassing both hospital and community-acquired infections. While these bacteria deploy numerous virulence factors in the initiation, persistence, and dissemination of infections, antibiotic resistance stands as a formidable attribute enhancing their survival and success. This study aimed to characterize the *S. aureus* isolates by whole genome sequence and determine their quinolone resistance.

A nearly equal proportion of MRSA (49.3%) and MSSA (50.7%) strains was found among the isolates. This MRSA proportion aligns with previous studies conducted in Moshi, Tanzania (43.3%), and among patients and healthcare workers in Uganda [24, 31]. Nonetheless, the aforementioned proportion is consistent with MRSA prevalence in several African countries [6] but higher than a study in Libya with a detected prevalence of 16% [32]. The disparity can be attributed to sample size, demographics, infection control measures, and number of hospitals involved. MRSA strains are well known for their increased invasive ability, virulence, and resistance, which prolong hospital stays and treatment costs. There is an urgent need for an effective and sustainable surveillance system to monitor MRSA-associated infections in hospitals and communities. The study also observed high prevalence 61 (43.6%) of *S. aureus* in surgical wards. However, *S. aureus* is a common pathogen known to cause surgical site infections (SSIs) in surgical wards [33]. Considering the proportion of MRSA among the isolates, a high prevalence increases the likelihood of postoperative infections which may associate with worse outcome including higher morbidity. This signals the need for immediate interventions in clinical practice and infection control protocols to prevent infections and reduce the risk spreading of resistant strains.

Most MRSA isolates (82.6%) in our study demonstrated resistance to quinolones (ciprofloxacin/Nalidixic acid). However, none of the isolates from MSSA demonstrated resistance to quinolones. The observed resistance to quinolones in MRSA was found to be mediated by a combination of single-point mutations in two non-resistance genes, *grlA* (p.S80Y) and *gyrA* (p.S84L). These genes encode for two replicating enzymes, topoisomerase IV and DNA gyrase, respectively [34, 35]. The point mutation in the *grlA* gene took place at position 80, replacing the amino acid serine with tyrosine, whereas the point mutation in *gyrA* took place at position 84, replacing serine with leucine. The observed mutations were simultaneously found to occur in all MRSA isolates that exhibited resistance to quinolones. It is worth noting that a high level of quinolone resistance results from the observed combination of point mutations [36, 37]. Mutations in *gyrB*/*gyrB* are also known to confer quinolone resistance of low levels [37]. However, no point mutation in either *gyrB* or *gyrB* genes was found in our study. Since quinolone resistance was observed in the majority of MRSA isolates in our study, suggesting that the use of quinolones for MRSA-related infections may need to be considered. Unfortunately, minimum inhibitory concentration (MIC) testing for quinolones was not conducted in this study. MIC data could have provided valuable insights by correlating genotypic antibiotic resistance findings with phenotypic resistance profiles, representing a potential limitation of this study.

Multilocus sequence typing identified 14 unrelated sequence types among the 140 *S. aureus* isolates, with ST8 being dominant among MRSA and ST152 among MSSA. This diversity suggests epidemiological unrelatedness between MRSA and MSSA. The SCC*mec* elements harbored in MRSA were further typed, and all isolates were observed to harbor mec elements type IV. Further genotyping regarding *Staphylococcus* protein A (*Spa*) also revealed diverse numbers, however, *spa* type 1476 and 355 were predominantly found in MRSA isolates and MSSA, respectively. This makes the overall common MRSA genotype of ST8-SCCmecIV-spa 1476, which is a typical genotype of community-acquired MRSA (CA-MRSA) as reported in other studies [38, 39]. A study conducted in Tanzania among HIV patients identified *spa* type 1476 ST8 and SCC*mec* type IV as the predominant strains [40]. The predominance of these Community-acquired *S. aureus* (CA-MRSA) isolates raises concerns about its prevalence in hospital settings. This highlights the urgency of controlling its spread and suggests that Community-acquired methicillin-resistant *S. aureus* (CA-MRSA) may eventually replace Hospital-acquired MRSA. These results are in line with a similar study conducted in Dar-es-salaam, Tanzania [40], but contrary to a study conducted in Eastern Tanzania [41]. The discrepancy can be explained by differences in study sites and the nature of samples sequenced [13].

High genetic diversity among the isolates was observed among isolates, particularly those from Dodoma Regional Referral Hospital and likewise from Tabora Regional Referral Hospital. This is evident from the high SNP difference of 1,3539 detected among the MRSA isolates. The significant genetic diversity of *S. aureus* allows them to survive and adapt to a wide range of environmental conditions. Nevertheless, despite the high genetic diversity observed, some isolates have been found to form clusters, suggesting they are closely related and share a common ancestor (Fig. 5). Most of the MRSA isolates from Mnazi Mmoja Referral Hospital were observed to exhibit closer genetic relatedness, suggesting they share a common ancestor between them. In this instance, a standalone cluster made up of 7 isolates with *SCCmec*IV-*spa*-t498 genotype with minor SNPs difference of 0 to 9 between them (FFZB_126, FFZB_79, FFZB_189, FFZB_154, FFZB_64, FFZB_48, and FFZB_201). It is worth considering that these seven isolates were collected from patients enrolled in different wards with the Surgical ward being the predominant one. Moreover, the same scenario has been observed among isolates from Morogoro Hospital, with four clusters found to exist among the isolates. It is also worth considering that the isolates forming all of the above-mentioned clusters were observed to comprise MRSA with genotype ST8-*SCCmec*IV-*sp*a-t1476. Notably, a subset of MRSA isolates all originating from Mnazi Mmoja Hospital exhibited the genotype of ST8- SCC*mec*IV*-sp*a-t498. The high genetic diversity reported in this study aligns with other reported in other studies done in Tanzania, which reported high genetic diversity from the hospital’s *S. aureus* isolates and among the HIV patients [24, 40]. Since isolates within most of these formed clusters had the same genotypic characteristics, and the findings also indicate that these clones are community-acquired, clonal transmission may have been a result of human movement.

The genotypic characterization of *S. aureus* isolates revealed distinct relationships between sequence types (STs), spa types, SCCmec types, and virulence factors. ST8 was the most prevalent and exclusively associated with MRSA, typically harboring SCCmec type IV and spa type t1476. These isolates exhibited a variety of resistance genes, including mecA, and were linked to healthcare-associated infections. In contrast, ST152, associated with spa type t355, was predominantly MSSA and displayed lower resistance profiles, indicating a potential for treatment with methicillin-based therapies. Notably, virulence factors such as the Panton-Valentine Leucocidin (PVL) gene were detected in both MRSA and MSSA isolates, suggesting that virulence and resistance traits may coexist independently. The acquisition of SCCmec IV in some ST88 isolates further highlights the genetic diversity and adaptability of *S. aureus* in acquiring resistance and virulence elements. The relationships between ST, spa types, SCCmec types, and virulence genes in your study reveal a complex interaction where certain genetic markers (such as ST8, SCCmec IV, and spa type t1476) are strongly associated with MRSA strains and the presence of resistance genes, including *mecA*, *ermC*, and *tet(K).* Conversely, ST152 and its associated spa type t355 predominantly represent MSSA strains, which are more susceptible to antibiotics [42]. The presence of virulence genes like PVL across both MRSA and MSSA strains further highlights that antibiotic resistance and virulence are not always linked but can coexist, complicating treatment strategies [5, 24]. The combination of ST8 and SCCmec IV in PVL-positive isolates suggests a heightened risk for severe infections due to the convergence of both resistance and virulence traits.

Aside from the *mecA* gene, several other resistance genes such as *blaZ*, *dfrG*, *aac (6’)-aph (2’’)*, and *ermC tet(K)*, were found distributed among the isolates. However, the majority of these resistance genes were more frequently observed in MRSA compared to MSSA. Intriguingly, numerous combinations of resistance genes were observed within the isolates, with a significant proportion exhibiting co-occurrence of two or more genes. Only 12% of isolates carried *blaZ* as the sole resistance determinant. Prominent combinations included *blaZ*-*dfrG*; *aac (6’)-aph (2’’)*-*blaZ*-*dfrG*-*ermC*-*mecA*; and *aac (6’)-aph (2’’)*-*blaZ*-*dfrG*-*ermC*-*mecA*-*tet(K)*, accounting for 20.7%, 17% and 10% respectively. The latter two combinations were exclusively identified in MRSA isolates, while the first combination was solely observed in MSSA isolates. Of more concern, the majority of these genes confer resistance against non-β-lactams drugs to readily β-lactams resistant MRSA isolates, thus further narrowing the treatment options. Curiously, no vancomycin resistance genes were found in studied isolates, offering hope for treatment as vancomycin remains the primary reserved antibiotic for treating MRSA in Tanzania and other developing countries. This underscores the urgent need for strong antimicrobial stewardship and preserves effective antibiotics.

### Conclusion

This study reveals that the prevalence of MRSA in Tanzania is increasing, with most isolates possessing extended antibiotic resistance, mediated by the vast array of resistance genes, including to quinolones, which are one of the commonly prescribed and affordable classes of antibiotics. This profoundly lowers therapeutic options and prompts the use of last-resort antibiotics such as colistin, which are less affordable and often accompanied by many side effects and toxicity. The escalation of antibiotic resistance, governed by the acquisition of diverse resistance genes as evident from this study, underscores the potential risk of emerging pan-resistant MRSA strains, which exhibit resistance to all commonly used antibiotics in Tanzania. There is a high genetic diversity among the *Staphylococcus aureus* isolates in regional referral hospitals in Tanzania, which highlights their genetic resilience, persistence, and adaptation.

### Recommendations

Constant vigilance is important in protecting our last-resort antibiotics from abuse in both hospitals and community settings. This is to prevent the depletion of functional antibiotics in our arsenals to fight off multidrug-resistant bacteria. Since the emergence of new drug resistance currently outpaces the development of new antibiotic agents, there is a high need to reduce antibiotic dependency through implementing infection control strategies. There is an urgent need for continued surveillance to combat MRSA and related escalating antibiotic resistance by reducing empirical use of antibiotics through screening for drug resistance before prescription, as well as heightening antimicrobial stewardship in over-the-counter sales. Hospital authorities should apply measures to reduce MRSA spread in communities and hospitals, introduce proper sanitation and disinfection of the hospital environment, and heighten screening and surveillance of MRSA in hospitals.

## Data Availability

The reads and genome assemblies for this study have been deposited in the European Nucleotide Archive under accession number PRJEB71932.
